# CLCA2 as a Novel Immunohistochemical Marker for Differential Diagnosis of Squamous Cell Carcinoma from Adenocarcinoma of the Lung

**DOI:** 10.1155/2014/619273

**Published:** 2014-12-07

**Authors:** Kazuya Shinmura, Hisaki Igarashi, Hisami Kato, Yuichi Kawanishi, Yusuke Inoue, Satoki Nakamura, Hiroshi Ogawa, Takashi Yamashita, Akikazu Kawase, Kazuhito Funai, Haruhiko Sugimura

**Affiliations:** ^1^Department of Tumor Pathology, Hamamatsu University School of Medicine, 1-20-1 Handayama, Higashi-ku, Hamamatsu, Shizuoka 431-3192, Japan; ^2^Research Equipment Center, Hamamatsu University School of Medicine, Hamamatsu 431-3192, Japan; ^3^Department of Internal Medicine 2, Hamamatsu University School of Medicine, Hamamatsu 431-3192, Japan; ^4^Division of Pathology, Seirei Mikatahara General Hospital, Hamamatsu 433-8558, Japan; ^5^Department of Surgery 1, Hamamatsu University School of Medicine, Hamamatsu 431-3192, Japan

## Abstract

Recent progress in targeted therapy for lung cancer has revealed that accurate differential diagnosis between squamous cell carcinoma (SCC) and adenocarcinoma (ADC) of the lung is essential. To identify a novel immunohistochemical marker useful for differential diagnosis between the two subtypes of lung cancer, we first selected 24 SCC-specific genes and 6 ADC-specific genes using data (case number, 980) from the Cancer Genome Atlas (TCGA) database. Among the genes, we chose the *CLCA2* gene, which is involved in chloride conductance and whose protein expression in lung cancer is yet to be characterized, and evaluated its protein expression status in 396 cases of primary lung cancer at Hamamatsu University Hospital. Immunohistochemical analysis revealed a significantly higher CLCA2 expression level in the SCCs than in the ADCs (*P* < 0.0001) and also a significantly higher frequency of CLCA2 protein expression in the SCCs (104/161, 64.6%) as compared with that in the ADCs (2/235, 0.9%) (*P* < 0.0001; sensitivity 64.6%, specificity 99.1%). The CLCA2 protein expression status was associated with the histological tumor grade in the SCCs. These results suggest that CLCA2 might be a novel excellent immunohistochemical marker for differentiating between primary SCC and primary ADC of the lung.

## 1. Introduction

Squamous cell carcinoma (SCC) and adenocarcinoma (ADC) are the two most common histological subtypes of lung cancer [1, 2]. Recent advances in the elucidation of the genetic alterations in lung cancers and in the chemotherapy for lung cancer patients have revealed differences in the most suitable chemotherapeutic regimens between SCC and ADC [3–5]. For example, bevacizumab, a monoclonal antibody directed against VEGF, and pemetrexed, a folate antimetabolite, are not used in SCC patients owing to an increased risk of fatal pulmonary hemorrhage and lack of effectiveness, respectively [6, 7]. In addition, erlotinib and gefitinib, small-molecule EGFR inhibitors, are only indicated in* EGFR* mutation-positive lung cancer, most cases of which are ADCs [8]. Thus, it is important to differentiate between SCC and ADC for assigning lung cancer patients to histology-based therapies.

In addition to light-microscopic examination of conventional hematoxylin and eosin stained sections, immunohistochemistry is often used to assist in various kinds of differential diagnosis [9, 10]. In regard to the differential diagnosis between lung SCC and ADC, for example, thyroid transcription factor-1 (TTF-1) has been identified as a specific marker of ADC, while p63, p40, and cytokeratin 5/6 (CK5/6) are known as SCC-specific markers [11, 12]. Immunohistochemical analysis using antibodies to these proteins is widely performed; however, the results of the analysis are not always reliable, because a subset of ADCs that are immunohistochemically positive for p63, p40, or CK5/6 and a subset of SCCs that are positive for TTF-1 have also been reported [11, 13–17]. To measure the mRNA expression level, microarray analysis has been used for a long time in spite of some limitations such as its low dynamic range and the occurrence of hybridization artifacts [18, 19]. Recently, however, the RNA-sequencing (RNA-seq) method has been developed, in which the aforementioned limitations of microarray analysis have been overcome; furthermore, this technique is also superior in terms of some issues such as the dynamic range, detection of low abundance transcripts, and differentiation of isoforms [20]. Thus, use of the RNA-seq method might be useful for the identification of novel alterations of mRNA expression in human cancers; however, only one report of comparison between SCC and ADC of the lung using RNA-seq expression data has been published, in which 88 lung cancer cases were analyzed [21]. Therefore, to identify novel reliable immunohistochemical markers useful for distinguishing between SCC and ADC of the lung, in this study, we screened whole genes by comparing the expression values derived from the processed RNA-seq data between 490 cases of SCC and 490 cases of ADC from the Cancer Genome Atlas (TCGA). Then from a total of 30 genes selected by the screening, we chose CLCA2 (chloride channel accessory 2), examined its expression status in 396 lung cancers, and found that CLCA2 is a sensitive and specific marker of SCC. Our study suggests that evaluation of the expression of CLCA2 is of value in distinguishing between SCC and ADC of the lung.

## 2. Materials and Methods

### 2.1. Collection of Publicly Available Gene Expression and Somatic Mutation Data

Gene expression data for 980 lung ADC and SCC cases and somatic mutation data for 173 lung SCC cases (TCGA public data available in April 2014) were collected from the TCGA data portal (https://tcga-data.nci.nih.gov/tcga/). The expression data were obtained as processed RNA-seq data in the form of RNA-seq by Expectation Maximization (RSEM) [22]. The somatic mutation data were obtained in the form of the mutation annotation format (MAF) file.

### 2.2. Preparation of Tissue Microarray (TMA) Blocks

Paraffin-embedded tissue samples from 235 cases of ADC of the lung and 161 cases of SCC of the lung who had undergone surgery at Hamamatsu University Hospital were used for the TMA block. To explain in further detail, the block was prepared by transferring a cylinder of 3 mm diameter from each of the paraffin-embedded tissue samples using a microarrayer (KIN-1, Azumaya, Tokyo, Japan), as previously described [23]. The histopathological diagnosis of lung cancer was confirmed by two pathologists (Kazuya Shinmura and Haruhiko Sugimura). This study was conducted with the approval of the Institutional Review Board (IRB) of Hamamatsu University School of Medicine.

### 2.3. Immunohistochemical Staining

Sections of TMA blocks were used for immunohistochemical staining with an automatic immunohistochemical stainer, the HISTOSTAINER (Nichirei Bioscience, Tokyo, Japan). To explain in greater detail, the sections were deparaffinized, rehydrated, and boiled at 96°C for 40 min in TE solution (pH 9.0) for antigen retrieval. Endogenous peroxidase activity was blocked by incubation for 5 min in a 3% hydrogen peroxide solution. Next, the sections were incubated with a rabbit anti-CLCA2 polyclonal antibody (Sigma, St. Louis, MO, USA) at a dilution of 1 : 1,000 for 30 min at room temperature (RT). After washing, the sections were incubated for 30 min at RT with an amino acid polymer conjugated with goat anti-rabbit IgG and horseradish peroxidase (Histofine Simple Stain MAX-PO kit, Nichirei, Tokyo, Japan). The antigen-antibody complex was visualized with 3,3′-diaminobenzidine tetrahydrochloride, and the sections were counterstained with hematoxylin. Esophageal stratified squamous epithelium and gastric foveolar epithelium were used as positive control and negative control, respectively, for the CLCA2 immunohistochemistry (see Supplementary Figure S1 in Supplementary Material available online at http://dx.doi.org/10.1155/2014/619273), based on previous reports [24].

### 2.4. Immunohistochemistry Scoring

The staining intensities of the cancer cells for CLCA2 were graded on a four-point scale as follows: 0 (blue), 1+ (blue/brown), 2+ (brown), and 3+ (bright brown). The percentage of cells with each intensity value was then multiplied by the intensity value, to obtain an immunohistochemical score of 0–300. The immunohistochemical score was then classified as negative (0–100) or positive (101–300).

### 2.5. Statistical Analysis

The statistical analysis was performed using an unpaired *t*-test, Mann-Whitney *U* test, or chi-square test. Overall survival curves were constructed using the Kaplan-Meier method, and the log-rank test was used to evaluate the differences in the curves. The JMP version 9.0 software (SAS Institute, Cary, NC, USA) was used for the analyses. *P* values of less than 0.05 were considered to indicate statistical significance.

## 3. Results

### 3.1. Identification of Genes Specifically Expressed in SCC of the Lung as Compared to Those Expressed in ADC of the Lung or Vice Versa

**Figure 1:**
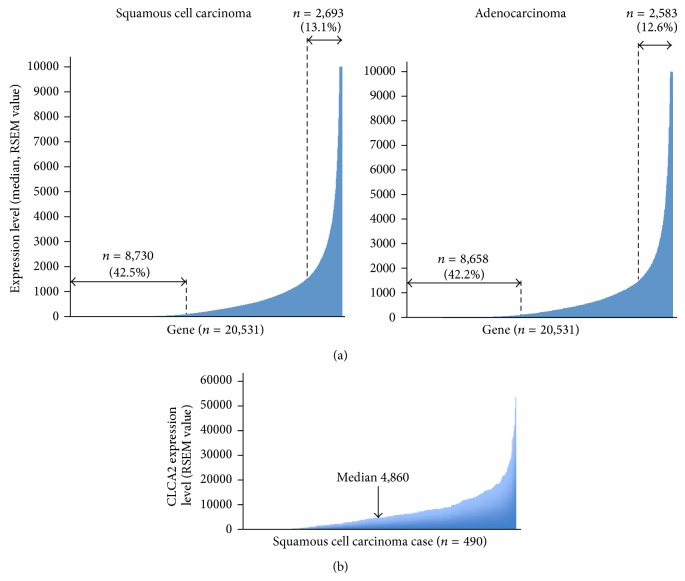
mRNA expression levels of whole genes in squamous cell carcinoma (SCC) and adenocarcinoma (ADC) of the lung using data from the TCGA database. (a) Median mRNA expression values of whole genes (*n* = 20, 531) in SCC (*n* = 490, left panel) and ADC (*n* = 490, right panel) of the lung. The expression levels are shown as RSEM values. Number and percentage of genes whose median expression values in cancer were more than 1,500 or less than 100 are shown. (b) CLCA2 mRNA expression level in SCCs of the lung (*n* = 490). The median expression value was 4,860.

To identify markers to differentiate between SCC and ADC of the lung by immunohistochemistry, we first attempted to utilize the mRNA expression data, based on the RNA-seq experiment, of cases of SCC (*n* = 490) and ADC (*n* = 490) of the lung derived from the TCGA database. To identify SCC-specific genes, we selected genes satisfying the following two conditions: (1) median gene expression value more than 1,500 in SCC specimens and (2) median gene expression value less than 100 in ADC specimens. A total of 2,693 genes (13.1%) conformed to condition (1) and a total of 8,658 genes (42.2%) conformed to condition (2), and 24 genes satisfied both conditions (1) and (2) and were considered as SCC-specific genes (Figure 1(a) and Table 1). The 24 genes included previously identified SCC-specific genes such as* KRT5*,* KRT6*,* TP63*,* DSC3*, and* KRT14* [16, 25, 26]. Next, to identify ADC-specific genes we selected genes satisfying the following two conditions: (1) median gene expression value more than 1,500 in the ADC specimens and (2) median gene expression value less than 100 in the SCC specimens. A total of 2,583 genes (12.6%) conformed to condition (1) and a total of 8,730 genes (42.5%) conformed to condition (2) and 6 genes satisfied both conditions (1) and (2) and were considered as ADC-specific genes (Figure 1(a) and Table 2). Among them was the* NKX2-1* (alternative name* TTF-1*) gene, which is known as an ADC-specific marker, and the* LMO3* gene, one of the transcriptional targets of* NKX2-1* [11, 27]. These results indicate that our selection based on the comparison of the median expression values of whole genes in SCCs and ADCs of the lung was valid to identify candidate genes for differentiating between SCC and ADC of the lung.

### 3.2. CLCA2 Protein Is Specifically Expressed in SCC of the Lung

**Figure 2:**
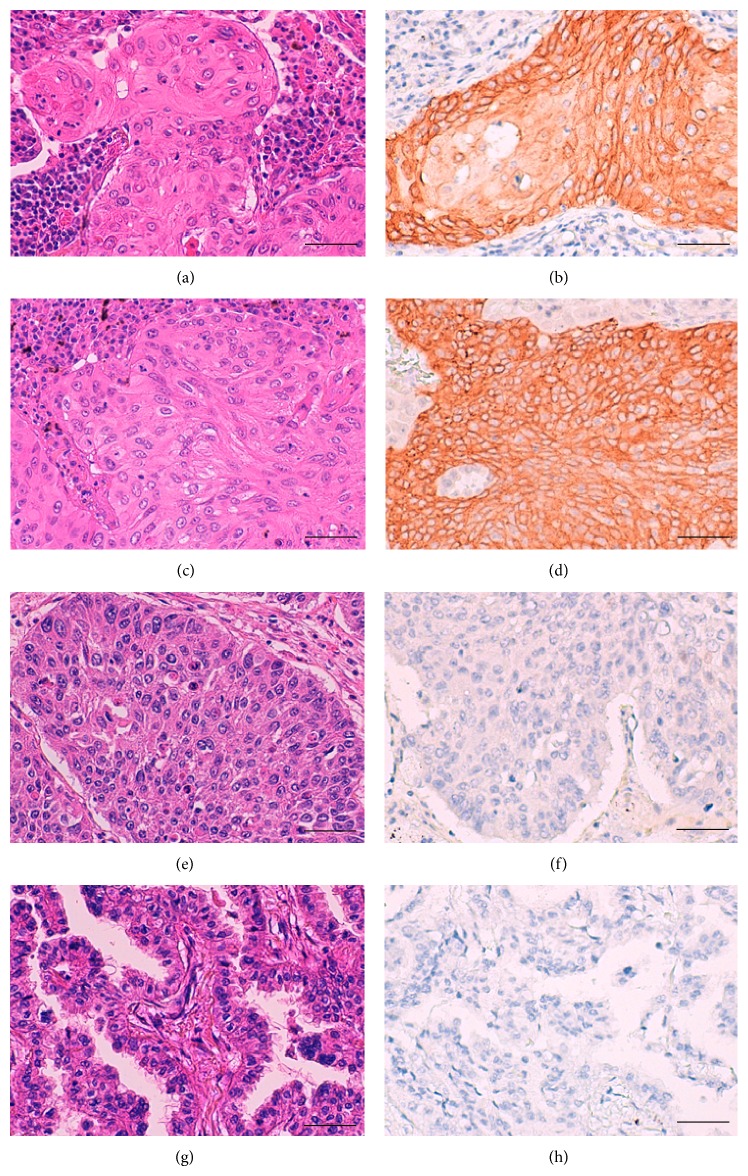
Examples of CLCA2 immunoreactivity in squamous cell carcinoma (SCC) and adenocarcinoma (ADC) of the lung. Positive immunohistochemical staining for CLCA2 was observed in two SCC cases ((a) and (c) Hematoxylin and eosin (H&E); (b) and (d) CLCA2 immunohistochemistry), and negative staining was observed in one SCC case ((e) H&E; (f) CLCA2 immunohistochemistry) and one ADC case ((g) H&E; (h) CLCA2 immunohistochemistry). Scale bar = 50 *μ*m.

**Figure 3:**
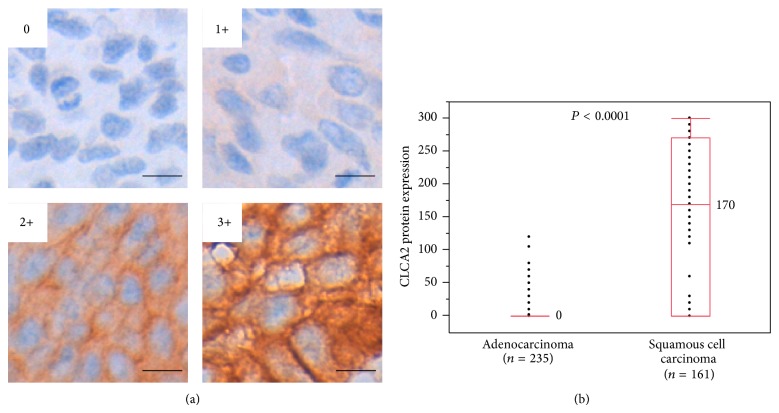
CLCA2 protein expression level in adenocarcinoma (ADC) and squamous cell carcinoma (SCC) of the lung. (a) The staining intensity of the cancer cells in immunohistochemical analysis using anti-CLCA2 antibody. Examples for intensity levels of the values of 0, 1+, 2+, and 3+ are shown. Scale bar = 10 *μ*m. (b) Box-plot analysis was performed for the results of CLCA2 immunohistochemistry in cases with ADC (*n* = 235) and cases with SCC of the lung (*n* = 161). Median values are shown. A statistically significant difference in the CLCA2 expression level was detected between the two groups (*P* < 0.0001, Mann-Whitney *U* test).

**Figure 4:**
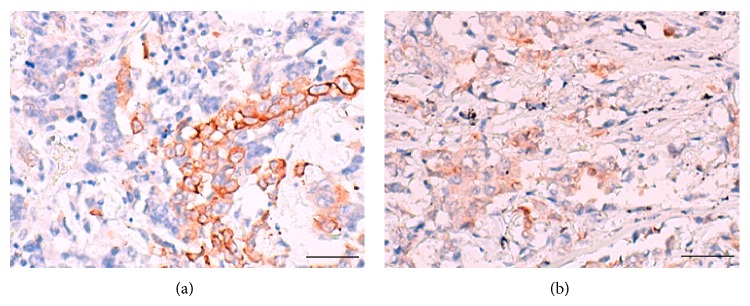
Examples of a rare case of adenocarcinoma (ADC) of the lung showing CLCA2 immunoreactivity. CLCA2 immunohistochemistry revealed partial staining in two cases of ADC ((a) and (b)). Scale bar = 50 *μ*m.

Among the genes listed in Tables 1 and 2, we focused on the* CLCA2* gene since its protein expression status in lung cancer has not been reported previously and we found a significant difference in the median expression value of this gene between the SCCs and ADCs (4,860.0234 versus 5.2242) (Table 1 and Figure 1(b)); furthermore, anti-CLCA2 antibody is available. To judge whether determination of the CLCA2 protein expression status might be useful for distinguishing between SCC and ADC, we performed an immunohistochemical analysis using anti-CLCA2 polyclonal antibody in TMA sections containing cancerous tissues in 235 specimens of primary ADC of the lung and 161 specimens of primary SCC of the lung (Hamamatsu University Hospital, Japan). CLCA2 immunoreactivity was observed in the cytoplasm and membrane of the cancer cells (Figure 2), and calculation of the immunohistochemical score revealed that it was significantly higher in the SCCs than in the ADCs (median, 170 versus 0; *P* < 0.0001, Mann-Whitney *U* test) (Figures 2 and 3). Moreover, when the immunohistochemical score of 100 was used as the cut-off value for defining positive CLCA2 expression (score > 101) and negative CLCA2 expression (score ≤ 100), positive expression was found at a significantly higher frequency in the SCCs (104/161 cases, 64.6%) as compared with that in the ADCs (2/235 cases, 0.9%) (*P* < 0.0001, chi-square test) (Table 3). The immunohistochemical scores in the two CLCA2-positive ADC cases were relatively low (120 and 105) (Figure 4). The sensitivity of measurement of CLCA2 expression for the diagnosis of SCC was 64.6%, and the specificity was 99.1% (Table 3). In the noncancerous lung, the bronchial epithelium and alveolar epithelium were negative to very weakly positive for CLCA2 expression (Supplementary Figure S2). Thus, the results suggest that CLCA2 may be a novel immunohistochemical marker for differentiating between SCC and ADC of the lung.

### 3.3. Association of the CLCA2 Protein Expression Status and Clinicopathological Factors in SCC of the Lung

**Figure 5:**
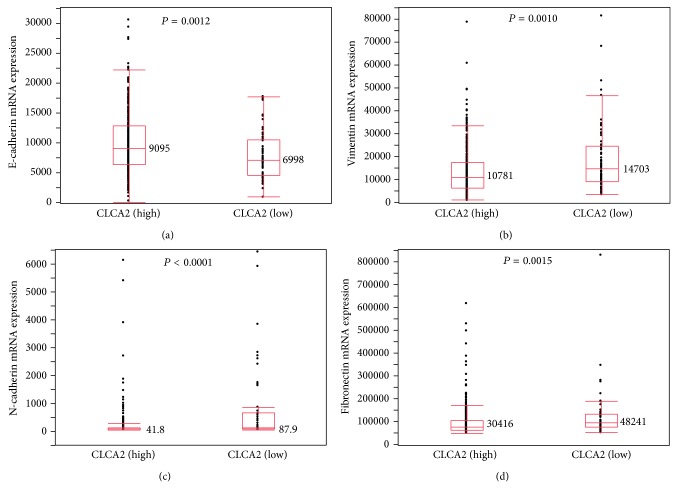
Comparison of the mRNA expression profile of the epithelial to mesenchymal transition marker between groups showing high and low CLCA2 expression levels using data from the TCGA database. The SCC patients were divided into a high CLCA2 expression group (RSEM values > 100; *n* = 429) and low CLCA2 expression group (RSEM values ≤ 100; *n* = 61). Statistically significant differences in the expression levels of E-cadherin (a), vimentin (b), N-cadherin (c), and fibronectin (d) were detected between the groups (Mann-Whitney *U* test). Median values are shown.

We next examined whether CLCA2 expression might be associated with any clinicopathological factors in SCC of the lung. Although no associations were found between the clinicopathological factors of gender, age, pT stage, or pN stage and the CLCA2 protein expression status, the frequency of cancers showing poorer differentiation (G3) was higher in the CLCA2-negative cancers than in the CLCA2-positive cancers (*P* < 0.0001, chi-square test) (Table 4). Since it has recently been revealed that loss of CLCA2 promotes the epithelial to mesenchymal transition (EMT) in breast cancer cells [28], we next examined whether the transcriptional profile of EMT markers might be changed in SCC of the lung using data from the TCGA database. The low CLCA2 expression group (RSEM expression value ≤ 100) exhibited significantly decreased E-cadherin expression (*P* = 0.0012, Mann-Whitney *U* test) and significantly increased vimentin (*P* = 0.0010), N-cadherin (*P* < 0.0001), and fibronectin (*P* = 0.0015) expressions as compared to the high CLCA2 expression group (RSEM expression value > 100); all of the results are compatible with the EMT (Figure 5). Next, by using both the expression and somatic mutation data from the TCGA database, we examined whether the CLCA2 somatic mutation status might be correlated with the CLCA2 expression level in SCC of the lung. Four SCC cases with a missense mutation (2.3%) and one SCC case with a nonsense mutation (0.6%) were observed among a total of 173 SCC cases (Table 5). The former cases showed high CLCA2 mRNA expression levels and the latter showed relatively low CLCA2 expression level (Table 5), although the difference was not statistically significant. All the above results suggested that the CLCA2 expression status was associated with the tumor grade and changes in the status of expression of the EMT markers in SCC of the lung.

### 3.4. Impact of the Difference in the CLCA2 Expression Level on the Survival in Patients with Lung SCC

**Figure 6:**
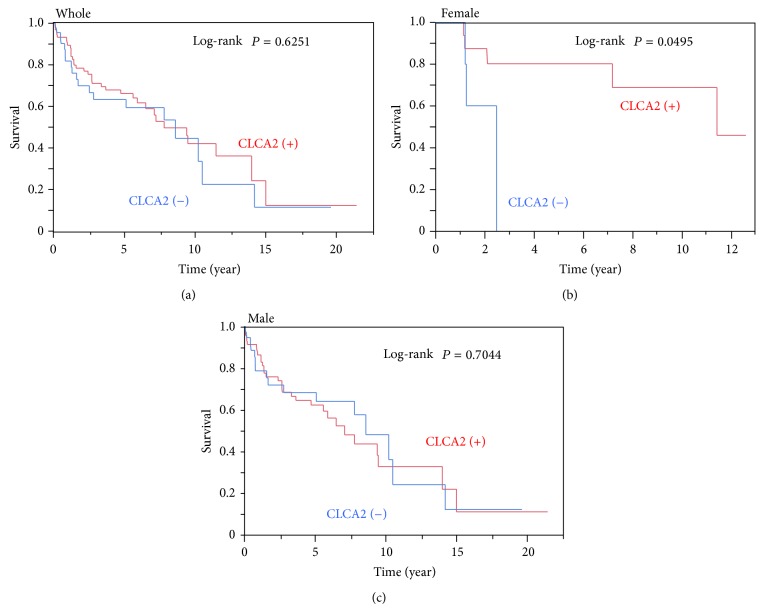
Impact of the CLCA2 expression level on the overall survival in patients with squamous cell carcinoma (SCC) of the lung. The survival curves in patients with SCC of the lung from the Hamamatsu University Hospital were generated using the Kaplan-Meier method. (a) The patients with SCC of the lung (*n* = 138) were divided into a CLCA2 protein-positive group (red) and CLCA2 protein-negative group (blue), and the overall survival rates in the two groups were compared. ((b) and (c)) Female patients (*n* = 26) (b) or male patients (*n* = 112) (c) with lung SCC were divided into a CLCA2 protein-positive group (red) and CLCA2 protein-negative group (blue), and the overall survival rates in the two groups were compared.

Finally, we examined whether the CLCA2 protein expression status might be predictive of the prognosis in patients with lung SCC. Kaplan-Meier analysis for survival of the data of 138 SCC patients included in the CLCA2 protein expression analysis revealed no statistically significant difference in the overall survival (Figure 6(a)). However, when the SCC patients were divided into male and female groups, the prognosis of the patients with negative CLCA2 expression was significantly poorer than that of those exhibiting positive CLCA2 expression in the female patient group (*n* = 26; log-rank *P* = 0.0495) (Figure 6(b)), but not in the male patient group (*n* = 112) (Figure 6(c)). These results suggested that negative CLCA2 expression was associated with a poorer survival in female patients with SCC of the lung.

## 4. Discussion

In this study, 24 SCC-specific genes and 6 ADC-specific genes at the mRNA expression level were identified using data from the TCGA database. Among the selected genes, the* CLCA2* gene, which is involved in chloride conductance, was selected and its protein expression status was evaluated in a total of 396 cases of primary lung cancer from Hamamatsu University Hospital. Immunohistochemical analysis revealed that the CLCA2 protein expression level was significantly higher in the SCCs than that in the ADCs and that positive CLCA2 protein expression occurred at a significantly higher frequency in SCC of the lung as compared with that in ADC of the lung (sensitivity 64.6%, specificity 99.1%). These results suggest that CLCA2 might be a novel immunohistochemical marker useful for the differential diagnosis between lung SCC and ADC.

The CLCA2 protein belongs to the calcium sensitive chloride conductance protein family and one of three CLCA proteins (CLCA1, CLCA2, and CLCA4) [29–31]. It has been reported that CLCA2 mRNA is more strongly expressed in SCC of the lung as compared to that in ADC of the lung [32]; however, the CLCA2 protein expression status in lung cancer had not yet been investigated. Our current study clearly revealed, for the first time, that CLCA2 is a sensitive and specific immunohistochemical marker for SCC of the lung. The sensitivity of measurement of CLCA2 expression (64.6%) was relatively low as compared to that of measurement of p63, p40, and CK5/6 reported previously [16, 17, 33], whereas it was better than that for other SCC-specific markers, such as glypican-3 (46%), S100A2 (63%), and CD141 (46%) reported previously [16, 33]. In regard to the specificity of CLCA2 (99.1%), the specificity was superior to that for most of the known immunohistochemical markers of SCC (47 to 100%) [11, 13, 15, 16, 33]. Thus, we considered that CLCA2 as a marker of SCC has the advantage of high specificity. Combined use of CLCA2 with another SCC-specific marker in the future may be expected to increase the value of the marker as a diagnostic tool for distinguishing between SCC and ADC of the lung, similar to the combination of TTF-1 and napsin A for distinguishing between ADC and SCC of the lung, which is widely employed in practical diagnosis [34].

Loss of CLCA2 expression has been reported to be associated with poor clinical outcomes in patients with breast cancer [28]; however, the impact of reduced expression of CLCA2 on the survival in patients with other types of cancer has not yet been reported. Our current study revealed that loss of CLCA2 protein expression was a poor prognostic factor in female patients with lung SCC, indicating that the CLCA2 expression status is associated with a poor prognosis, not only in patients with breast cancer but also in those with SCC of the lung. CLCA2 is one of the targets of p53 [35–37] and CLCA2 negatively regulates proliferation, migration, and invasion of cancer cells [35, 36]. Moreover, reduced CLCA2 expression is associated with transcriptional changes of the EMT marker in SCC of the lung as shown in this study and in breast cancer as shown in a previous study [28]. Thus, acquisition of survival advantage of cancer cells associated with downregulation of CLCA2 might be one of the reasons why reduced CLCA2 expression leads to a poor survival outcome in patients with lung SCC. Interestingly, the phenomenon of reduced expression of a lung SCC-specific marker being associated with a poor clinical outcome has also been observed for the* DSC3* gene [38], suggesting that it may not be a rare event. In regard to the question of why loss of CLCA2 expression did not exert any significant impact on the survival outcome in male patients with SCC of the lung, we do not have satisfactory answer at present. On the other hand, several survival markers, such as abnormal expression of microRNA miR-183-3p, miR-122, and miR-195, are known in female lung cancer patients [39, 40]. In any case, the number of SCC cases analyzed for the survival outcome in this study was small (male, *n* = 112; female, *n* = 26); investigation of a larger number of SCC cases in the future might lead to a more definitive conclusion on the impact of the CLCA2 expression status on the prognosis in patients with SCC of the lung.

In this study, a total of 30 genes expressed differentially between SCC and ADC of the lung were identified by analysis of the RNA-seq expression data. In addition to the genes previously recognized as diagnostic markers and the* CLCA2* gene studied here, the selected genes also included several other genes that have not previously been characterized in regard to their usefulness as immunohistochemical markers. Since the nonavailability of reliable antibodies for immunohistochemical analysis is one of the main reasons why they have not been characterized so far, future progress in the development of antibodies for such proteins might lead to the identification of useful immunohistochemical markers. Moreover, since most genes occur in several isoforms, it is possible that the immunohistochemical results might differ depending on the isoform recognized by a particular antibody and lead to the identification of novel useful markers. For example, an anti-p40 antibody was found to be useful antibody specific for SCC through such trials [17]. Thus, comparative analyses in the future of various antibodies against the proteins encoded by each of the listed 30 genes are considered to be important.

At present, the physiological function of CLCA2 in the normal lung epithelium remains unclear. Our immunohistochemical analysis revealed a negative or very weakly positive result for the protein expression of CLCA2 in normal lung epithelium. In addition, according to the TCGA data, the mRNA expression level of CLCA2 in the normal lung is low (median RSEM values in the normal lung samples of two groups: 16.0419 and 34.3854). The low CLCA2 expression level in the normal lung suggests that the role of CLCA2 in the noncancerous lung tissue might not be greater than that in patients with squamous cell carcinoma of the lung. However, there are many important genes whose expression levels are low at physiological condition, and careful consideration of this point is needed. The cytoplasmic and/or membranous localization of CLCA2 detected in the lung cancer cells in the present study implies that cellular localization is convenient for potential expression of the physiological role of CLCA2 in the regulation of chloride conductance and cell adhesion.

In conclusion, 30 candidate genes that were specifically expressed depending on the lung cancer histology were selected, and, among these, we identified CLCA2 as a novel immunohistochemical marker useful for differential diagnosis between SCC and ADC of the lung. Furthermore, our results also suggested that loss of CLCA2 expression might be a marker of poor survival outcome in a subset of patients with SCC of the lung.

## Supplementary Material

Supplementary Figure S1: Positive and negative controls for CLCA2 immunohistochemistry
Supplementary Figure S2: CLCA2 immunoreactivity in non-cancerous bronchial epithelium and alveolar epithelium of the lung

## Figures and Tables

**Table 1:** Genes specifically expressed in squamous cell carcinoma of the lung.

Gene^a^		Median expression value^b^	
Symbol	Description	Squamous cell carcinoma	Adenocarcinoma
KRT5	Keratin 5	100708.3884	40.18975
KRT6A	Keratin 6A	72788.57475	62.8728
PKP1	Plakophilin 1 (ectodermal dysplasia/skin fragility syndrome)	17091.659	97.2654
TP63	Tumor protein P63	9662.86635	69.23865
KRT6B	Keratin 6B	9498.8982	11.0179
CALML3	Calmodulin-like 3	6967.225	1.4188
DSG3	Desmoglein 3	6477.94655	1.12715
DSC3	Desmocollin 3	6416.14155	9.26815
KRT16	Keratin 16	5794.0321	49.5683
CLCA2	Chloride channel accessory 2	4860.0234	5.2242
KRT6C	Keratin 6C	4570.2383	5.23245
NTRK2	Neurotrophic tyrosine kinase, receptor, type 2	3944.6804	44.23865
SERPINB5	Serpin peptidase inhibitor, clade B (ovalbumin), member 5	3622.93785	22.98405
KRT13	Keratin 13	3557.4447	5.47095
GPX2	Glutathione peroxidase 2 (gastrointestinal)	3533.0995	99.7255
KRT14	Keratin 14	3094.8684	5.4587
FAT2	FAT atypical cadherin 2	2831.3711	18.2264
UGT1A6	UDP glucuronosyltransferase 1 family, polypeptide A6	2363.18645	17.0277
ANXA8	Annexin A8	2293.52705	38.38905
PTPRZ1	Protein tyrosine phosphatase, receptor-type, Z polypeptide	1949.045	28.1362
AKR1B10	Aldo-keto reductase family 1, member B10 (aldose reductase)	1708.73775	26.0211
PTHLH	Parathyroid hormone-like hormone	1686.3337	38.97765
BMP7	Bone morphogenetic protein 7	1682.00915	40.95445
GBP6	Guanylate binding protein family, member 6	1522.3459	16.8889

^a^All the genes conforming to the following two conditions are listed: (1) median expression value of the gene ≥1500 in squamous cell carcinoma and (2) median expression value of the gene ≤100 in adenocarcinoma.

^b^The RSEM value is used to show the expression level.

**Table 2:** Genes specifically expressed in adenocarcinoma of the lung.

Gene^a^		Median expression value^b^	
Symbol	Description	Adenocarcinoma	Squamous cell carcinoma
NKX2-1 (TTF-1)	NK2 homeobox 1 (thyroid transcription factor 1)	2956.61025	82.2006
SFTA3	Surfactant associated 3	2412.19165	66.691
SFTA2	Surfactant associated 2	1951.85825	41.01335
LMO3	LIM domain only 3 (rhombotin-like 2)	1901.15055	95.90595
XAGE1D	X antigen family, member 1D	1873.0292	5.0326
CLDN3	Claudin 3	1647.12575	93.83215

^a^All the genes conforming to the following two conditions are listed: (1) median expression value of the gene ≥1500 in adenocarcinoma and (2) median expression value of the gene ≤100 in squamous cell carcinoma.

^b^The RSEM value is used to show the expression level.

**Table 3:** CLCA2 protein expression status in 161 primary squamous cell carcinomas of the lung and 235 primary adenocarcinomas of the lung.

Histology	CLCA2 protein expression			Sensitivity	Specificity
	Positive (*n* = 106)	Negative (*n* = 290)	*P* value^a^		
Squamous cell carcinoma (*n* = 161)	104 (64.6%)	57 (35.4%)	<0.0001	64.6%	99.1%
Adenocarcinoma (*n* = 235)	2 (0.9%)	233 (99.1%)			

^a^Chi-square test.

**Table 4:** CLCA2 protein expression status and clinicopathological factors in 161 patients with primary squamous cell carcinoma of the lung.

Factor	Number of patients	CLCA2 protein expression status by IHC analysis		
		Positive (*n *= 104)	Negative (*n *= 57)	*P* value
Gender				
Female	31	22 (71.0%)	9 (29.0%)	0.4091^b^
Male	130	82 (63.1%)	48 (36.9%)	
Age				
<60	29	16 (55.2%)	13 (44.8%)	0.2412^b^
60≤	132	88 (66.7%)	44 (33.3%)	
Average ± SD^a^	67.2 ± 8.7	68.0 ± 7.9	65.7 ± 10.0	0.1098^c^
Grading				
G1 or G2	115	86 (74.8%)	29 (25.2%)	<0.0001^b^
G3	46	18 (39.1%)	28 (60.9%)	
pT stage				
pT1	44	30 (68.1%)	14 (31.8%)	0.5596^b^
pT2–pT4	117	74 (63.3%)	43 (36.7%)	
pN stage				
pN0	97	66 (68.0%)	31 (32.0%)	0.3712^b^
pN1–pN3	59	36 (61.0%)	23 (39.0%)	

^a^SD: standard deviation. ^b^Chi-square test. ^c^ *t*-test.

**Table 5:** *CLCA2* somatic nonsynonymous mutation and CLCA2 mRNA expression level in primary squamous cell carcinoma of the lung (*n* = 173) using data from the TCGA database.

TCGA ID	Nonsynonymous CLCA2 mutation		CLCA2 expression^b^
	Nucleotide level^a^	Protein level	
TCGA-85-6561-01A-11D-1817-08	c.804G>C	p.Gln268His	6420.1162
TCGA-39-5019-01A-01D-1817-08	c.1357C>A	p.Leu453Met	7491.9606
TCGA-46-6025-01A-11D-1817-08	c.2453C>A	p.Ala818Asp	6539.875
TCGA-66-2793-01A-01D-1267-08	c.2695G>A	p.Asp899Asn	1119.2189
TCGA-66-2785-01A-01D-1522-08	c.1444A>T	p.Arg482Ter	140.5295

^a^The reference sequence is NM_006536.5.

^b^The RSEM value is used to show the expression level.

## References

[1] Bennett V. A., Davies E. A., Jack R. H., Mak V., Møller H. (2008). Histological subtype of lung cancer in relation to socio-economic deprivation in South East England. *BMC Cancer*.

[2] Travis W. D., Brambilla E., Riely G. J. (2013). New pathologic classification of lung cancer: relevance for clinical practice and clinical trials. *Journal of Clinical Oncology*.

[3] Cancer Genome Atlas Research Network (2012). Comprehensive genomic characterization of squamous cell lung cancers. *Nature*.

[4] Cancer Genome Atlas Research Network (2014). Comprehensive molecular profiling of lung adenocarcinoma. *Nature*.

[5] Pikor L. A., Ramnarine V. R., Lam S., Lam W. L. (2013). Genetic alterations defining NSCLC subtypes and their therapeutic implications. *Lung Cancer*.

[6] Scagliotti G., Hanna N., Fossella F., Sugarman K., Blatter J., Peterson P., Simms L., Shepherd F. A. (2009). The differential efficacy of pemetrexed according to NSCLC histology: a review of two phase III studies. *Oncologist*.

[7] Sandler A., Gray R., Perry M. C., Brahmer J., Schiller J. H., Dowlati A., Lilenbaum R., Johnson D. H. (2006). Paclitaxel-carboplatin alone or with bevacizumab for non-small-cell lung cancer. *The New England Journal of Medicine*.

[8] Pallis A. G., Syrigos K. N. (2013). Epidermal growth factor receptor tyrosine kinase inhibitors in the treatment of NSCLC. *Lung Cancer*.

[9] Kanakis D., Lendeckel U., Theodosiou P., Dobrowolny H., Mawrin C., Keilhoff G., Bukowska A., Dietzmann K., Bogerts B., Bernstein H.-G. (2013). ADAM 12: a putative marker of oligodendrogliomas?. *Disease Markers*.

[10] Barresi V., Ieni A., Branca G., Tuccari G. (2014). Brachyury: a diagnostic marker for the differential diagnosis of chordoma and hemangioblastoma versus neoplastic histological mimickers. *Disease Markers*.

[11] Mukhopadhyay S., Katzenstein A.-L. A. (2011). Subclassification of non-small cell lung carcinomas lacking morphologic differentiation on biopsy specimens: utility of an immunohistochemical panel containing TTF-1, napsin A, p63, and CK5/6. *The American Journal of Surgical Pathology*.

[12] Nobre A. R., Albergaria A., Schmitt F. (2013). P40: a p63 isoform useful for lung cancer diagnosis—a review of the physiological and pathological role of p63. *Acta Cytologica*.

[13] Camilo R., Capelozzi V. L., Coelho Siqueira S. A., del Carlo Bernardi F. (2006). Expression of p63, keratin 5/6, keratin 7, and surfactant-A in non-small cell lung carcinomas. *Human Pathology*.

[14] Matoso A., Singh K., Jacob R., Greaves W. O., Tavares R., Noble L., Resnick M. B., Delellis R. A., Wang L. J. (2010). Comparison of thyroid transcription factor-1 expression by 2 monoclonal antibodies in pulmonary and nonpulmonary primary tumors. *Applied Immunohistochemistry and Molecular Morphology*.

[15] Terry J., Leung S., Laskin J., Leslie K. O., Gown A. M., Ionescu D. N. (2010). Optimal immunohistochemical markers for distinguishing lung adenocarcinomas from squamous cell carcinomas in small tumor samples. *The American Journal of Surgical Pathology*.

[16] Tsuta K., Tanabe Y., Yoshida A., Takahashi F., Maeshima A. M., Asamura H., Tsuda H. (2011). Utility of 10 immunohistochemical markers including novel markers (desmocollin-3, glypican 3, S100A2, S100A7, and Sox-2) for differential diagnosis of squamous cell carcinoma from adenocarcinoma of the lung. *Journal of Thoracic Oncology*.

[17] Bishop J. A., Teruya-Feldstein J., Westra W. H., Pelosi G., Travis W. D., Rekhtman N. (2012). P40 (ΔNp63) is superior to p63 for the diagnosis of pulmonary squamous cell carcinoma. *Modern Pathology*.

[18] Wang Y., Li Y., Liu S., Shen W., Jiang B., Xu X., Xie Y. (2005). Study on the dynamic behavior of a DMA microarray. *Journal of Nanoscience and Nanotechnology*.

[19] Casneuf T., van de Peer Y., Huber W. (2007). In situ analysis of cross-hybridisation on microarrays and the inference of expression correlation. *BMC Bioinformatics*.

[20] Zhao S., Fung-Leung W.-P., Bittner A., Ngo K., Liu X. (2014). Comparison of RNA-Seq and microarray in transcriptome profiling of activated T cells. *PLoS ONE*.

[21] Han S. S., Kim W. J., Hong Y., Hong S. H., Lee S. J., Ryu D. R., Lee W., Cho Y. H., Ryu Y. J., Won J. Y., Rhee H., Park J. H., Jang S. J., Lee J. S., Choi C. M., Lee J. C., Lee S. D., Oh Y. M. (2014). RNA sequencing identifies novel markers of non-small cell lung cancer. *Lung Cancer*.

[22] Li B., Dewey C. N. (2011). RSEM: accurate transcript quantification from RNA-Seq data with or without a reference genome. *BMC Bioinformatics*.

[23] Shinmura K., Goto M., Suzuki M., Tao H., Yamada H., Igarashi H., Matsuura S., Maeda M., Konno H., Matsuda T., Sugimura H. (2011). Reduced expression of MUTYH with suppressive activity against mutations caused by 8-hydroxyguanine is a novel predictor of a poor prognosis in human gastric cancer. *Journal of Pathology*.

[24] Connon C. J., Yamasaki K., Kawasaki S., Quantock A. J., Koizumi N., Kinoshita S. (2004). Calciun-activated chloride channel-2 in human epithelia. *Journal of Histochemistry and Cytochemistry*.

[25] Wang B. Y., Gil J., Kaufman D., Gan L., Kohtz D. S., Burstein D. E. (2002). p63 in pulmonary epithelium, pulmonary squamous neoplasms, and other pulmonary tumors. *Human Pathology*.

[26] Chen Y., Cui T., Yang L., Mireskandari M., Knoesel T., Zhang Q., Pacyna-Gengelbach M., Petersen I. (2011). The diagnostic value of cytokeratin 5/6, 14, 17, and 18 expression in human non-small cell lung cancer. *Oncology*.

[27] Watanabe H., Francis J. M., Woo M. S. (2013). Integrated cistromic and expression analysis of amplified *NKX2-1* in lung adenocarcinoma identifies *LMO3* as a functional transcriptional target. *Genes & Development*.

[28] Walia V., Yu Y., Cao D., Sun M., McLean J. R., Hollier B. G., Cheng J., Mani S. A., Rao K., Premkumar L., Elble R. C. (2012). Loss of breast epithelial marker hCLCA2 promotes epithelial-to-mesenchymal transition and indicates higher risk of metastasis. *Oncogene*.

[29] Gruber A. D., Schreur K. D., Ji H.-L., Fuller C. M., Pauli B. U. (1999). Molecular cloning and transmembrane structure of hCLCA2 from human lung, trachea, and mammary gland. *American Journal of Physiology—Cell Physiology*.

[30] Elble R. C., Walia V., Cheng H.-C., Connon C. J., Mundhenk L., Gruber A. D., Pauli B. U. (2006). The putative chloride channel hCLCA2 has a single C-terminal transmembrane segment. *Journal of Biological Chemistry*.

[31] Yu Y., Walia V., Elble R. C. (2013). Loss of CLCA4 promotes epithelial-to-mesenchymal transition in breast cancer cells. *PLoS ONE*.

[32] Hayes D. C., Secrist H., Bangur C. S., Wang T., Zhang X., Harlan D., Goodman G. E., Houghton R. L., Persing D. H., Zehentner B. K. (2006). Multigene real-time PCR detection of circulating tumor cells in peripheral blood of lung cancer patients. *Anticancer Research*.

[33] Kim M. J., Shin H. C., Shin K. C., Ro J. Y. (2013). Best immunohistochemical panel in distinguishing adenocarcinoma from squamous cell carcinoma of lung: tissue microarray assay in resected lung cancer specimens. *Annals of Diagnostic Pathology*.

[34] Ye J., Findeis-Hosey J. J., Yang Q., McMahon L. A., Yao J. L., Li F., Xu H. (2011). Combination of napsin A and TTF-1 immunohistochemistry helps in differentiating primary lung adenocarcinoma from metastatic carcinoma in the lung. *Applied Immunohistochemistry and Molecular Morphology*.

[35] Walia V., Ding M., Kumar S., Nie D., Premkumar L. S., Elble R. C. (2009). hCLCA2 is a p53-inducible inhibitor of breast cancer cell proliferation. *Cancer Research*.

[36] Sasaki Y., Koyama R., Maruyama R., Hirano T., Tamura M., Sugisaka J., Suzuki H., Idogawa M., Shinomura Y., Tokino T. (2012). CLCA2, a target of the p53 family, negatively regulates cancer cell migration and invasion. *Cancer Biology and Therapy*.

[37] Tanikawa C., Nakagawa H., Furukawa Y., Nakamura Y., Matsuda K. (2012). CLCA2 as a p53-inducible senescence mediatorspi. *Neoplasia*.

[38] Cui T., Chen Y., Yang L., Mireskandari M., Knösel T., Zhang Q., Kohler L. H., Kunze A., Presselt N., Petersen I. (2012). Diagnostic and prognostic impact of desmocollins in human lung cancer. *Journal of Clinical Pathology*.

[39] Zhang H., Su Y., Xu F., Kong J., Yu H., Qian B. (2013). Circulating microRNAs in relation to EGFR status and survival of lung adenocarcinoma in female non-smokers. *PLoS ONE*.

[40] Xu F., Zhang H., Su Y., Kong J., Yu H., Qian B. (2014). Up-regulation of *microRNA-183-3p* is a potent prognostic marker for lung adenocarcinoma of female non-smokers. *Clinical and Translational Oncology*.

