# Supplementary material for: CLCA2 as a Novel Immunohistochemical Marker for Differential Diagnosis of Squamous Cell Carcinoma from Adenocarcinoma of the Lung
Source: Dis Markers. 2014 Dec 7;2014:619273. doi: 10.1155/2014/619273 (PMC4274868; doi:10.1155/2014/619273)

Supplementary Information

for

CLCA2 as a Novel Immunohistochemical Marker for Differential Diagnosis of Squamous Cell Carcinoma from Adenocarcinoma of the Lung

Supplementary Figures S1 and S2

Supplementary FIGURE S1:

Positive and negative controls for CLCA2 immunohistochemistry

Supplementary FIGURE S2:

CLCA2 immunoreactivity in non-cancerous bronchial epithelium and alveolar epithelium of the lung

LEGENDS FOR SUPPLEMENTARY FIGURES

Supplementary Figure S1: Positive and negative controls for CLCA2 immunohistochemistry. (A) Esophageal stratified squamous epithelium was used as positive control. (B) Gastric foveolar epithelium was used as negative control. Scale bar = 20 μm.

Supplementary Figure S2: CLCA2 immunoreactivity in non-cancerous bronchial epithelium and alveolar epithelium of the lung. The bronchial epithelium (A) and alveolar epithelium (B) were negative to very weakly positive for CLCA2 expression. Scale bar = 20 μm.

Supplementary Figure 1

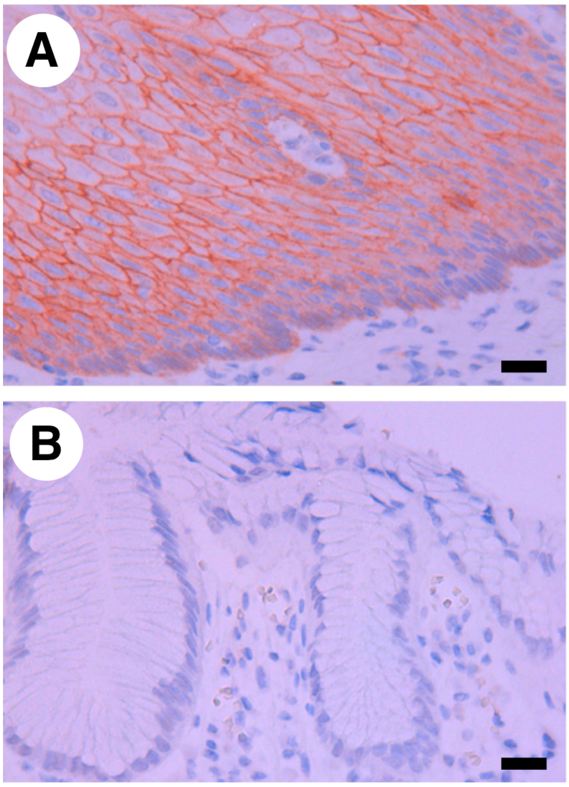

Supplementary Figure 2

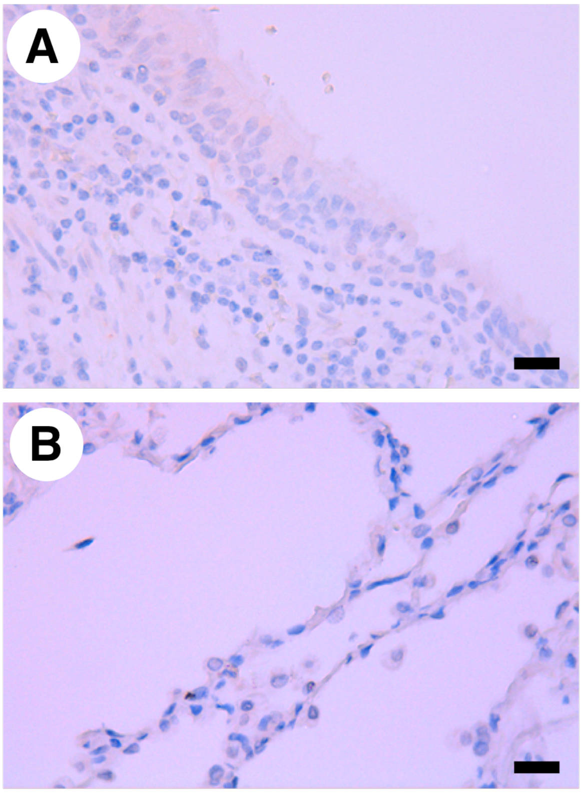

Supplement: Supplementary file 1 — Supplementary Figure S1: Positive and negative controls for CLCA2 immunohistochemistry Supplementary Figure S2: CLCA2 immunoreactivity in non-cancerous bronchial epithelium and alveolar epithelium of the lung [file 619273.f1.pdf]
